# Evolution and Resistance to Sexuality Education in Mexico

**DOI:** 10.9745/GHSP-D-17-00284

**Published:** 2018-03-21

**Authors:** Venkatraman Chandra-Mouli, Lucia Gómez Garbero, Marina Plesons, Iliana Lang, Esther Corona Vargas

**Affiliations:** a Department of Reproductive Health and Research/Human Reproduction Programme, World Health Organization, Geneva, Switzerland.; b School of Psychology, Universidad Catolica del Uruguay, Montevideo, Uruguay.; c School of Public Health, University of Washington, Seattle, WA, USA. Now with Department of Reproductive Health and Research/Human Reproduction Programme, World Health Organization, Geneva, Switzerland.; d Georgetown University, Washington, DC, USA.; e World Association for Sexual Health, Mexico City, Mexico.

## Abstract

**Background:**

Since the 1930s, Mexico has made substantial progress in providing adolescents with sexuality education through an evolving national school-based program. As part of a broader effort to document strategies to build support for and deal with resistance to sexuality education, this analysis uses a historical lens to answer 2 key questions: (1) How has the nature of sexuality education in Mexico evolved from the 1930s to the 2010s? (2) How have the drivers, responses, support, and resistance to sexuality education impacted Mexico's experience implementing and sustaining school-based sexuality education?

**Methods:**

The analysis was informed by a review of peer-reviewed and gray literature as well as the personal experience and documents of one of the authors, who has played a central role in Mexico's sexuality education effort for 50 years. The findings were organized according to 4 time periods—the 1930s, the 1970s, the 1990s, and the first 2 decades of the 21st century—that emerged during the analysis as distinct periods with regard to the social and political context of school-based sexuality education. Within each of these time periods, the following 4 thematic aspects were assessed: drivers, responses, support, and resistance, with a particular focus on the rationales and strategies of resistance over time.

**Findings:** This analysis identified determined support for school-based sexuality education in the 4 historical time periods from a range of governmental and nongovernmental stakeholders. However, opposition to sexuality education also steadily rose in the time period considered, with a growing range of more organized and well-financed actors. The Mexican government's commitment to delivering school-based sexuality education has driven its inclusion in public schools, along with expansion of its curricula from primarily biological content to a more comprehensive approach.

**Conclusion:**

Mexico's experience with sexuality education can inform other countries' efforts to consider the drivers, responses, support, and resistance that may be present in their own contexts. This type of analysis can contribute to strategic, well-informed, and well-conceived programmatic design and implementation to build support for sexuality education and deal with resistance.

## Abstract (precis)

Mexico's efforts at sexuality education have progressively evolved, from a biological focus in the socialist era in the 1930s, to adding a demographically concerned family planning component in the 1970s and including a wider reproductive health perspective in the 1990s, and finally shifting to a broader sociological context in the early 21st century. Opposition to sexuality education rose steadily in the time period considered, with a growing range of more organized and well-financed actors. Despite this opposition, alliances between academic, government, civil society, and NGO champions have helped ensure sustainability.

## INTRODUCTION

Over the past century, Mexico has made substantial progress in providing adolescents ages 10 to 19 years with sexuality education. First implemented in the 1930s, the government-run national school-based sexuality education program operates an integrated curriculum for primary and secondary school students. The current version of the national sexuality education program has been operational since the early 1970s. The program has experienced both support and resistance since its inception, with a notable evolution of its opposition's rationale and strategies over time. The strategies used by Mexico's governmental and nongovernmental advocates to maintain political commitment to sexuality education in the face of resistance can provide other countries with ideas and evidence to develop and support their own sexuality education programs.

Despite wide recognition of the need for sexuality education, evidence of its effectiveness in research and in certain countries, regional and national commitments to sexuality education, and availability of guidelines and programmatic resources for governments and NGOs, the United Nations Educational, Scientific and Cultural Organization (UNESCO) states that “there is less clarity about how to implement [sexuality education] and how to scale [it] up in diverse contexts,” especially when faced with sensitivity and resistance.^1^^–^^16^ Although Mexico's sexuality education program stands out due to its remarkable longevity, documentation of the program has thus far been limited. A review of existing evidence, including published and gray literature, reveals only brief references to the support of and resistance to sexuality education in Mexico, no analysis using a long-term perspective, and major gaps in the assessment of thematic components.^17^^–^^25^

Despite wide recognition of the need for sexuality education, “there is less clarity about how to implement [sexuality education] and how to scale [it] up in diverse contexts.”

To fill these gaps, this analysis aims to evaluate Mexico's experience with sexuality education as part of a broader effort by the World Health Organization (WHO) to document strategies to build support for sexuality education and deal with resistance in diverse contexts.^26^^,^^27^ This analysis will not restate the evidence base for sexuality education, nor will it assess the programs' coverage, quality, or fidelity, or the impact of the program on knowledge, behavioral, or health outcomes. Instead, this analysis will use a historical lens to answer the following questions:

1. How has the nature of sexuality education in Mexico evolved from the 1930s until the 2010s?
2. How have the drivers, responses, support, and resistance to sexuality education impacted Mexico's experience in implementing and sustaining school-based sexuality education?

## METHODS

### Data Collection

Data were collected through a literature review of peer-reviewed journals and national plans and reports, along with English and Spanish newspaper articles and website content to complement information gaps. The literature search was conducted using Google and Google Scholar with the following keywords: sex education, sexuality education, Mexico, history, and resistance. Mexico's government platform Gob.mx was searched for national plans and reports.^28^ After identifying specific organizations that opposed sexuality education during each time period, the researchers analyzed the rationale and strategies using information from each organization's website, as published sources were not available.^29^^–^^31^ Additionally, the personal experience and collection of documents of one of the authors (ECV), who has played a central role in Mexico's sexuality education efforts for more than 50 years, provided crucial information for understanding the challenges and successes of the program since the 1970s.

### Data Analysis

Relevant information was extracted from peer-reviewed and gray literature, including national plans and reports, newspaper articles, website content, and personal testimonies. The findings were organized according to 4 key time periods—the 1930s, the 1970s, the 1990s, and the first 2 decades of the 21st century—that emerged during the analysis as distinct periods with regard to the social and political context of school-based sexuality education. Within each of these time periods, the following 4 thematic aspects and questions were assessed:

1. Drivers: What was the social and political context that encouraged the delivery of school-based sexuality education?
2. Responses: How did sexuality education evolve in this time period?
3. Support: Who were the main players behind sexuality education's accomplishments?
4. Resistance: Who were the main players in the opposition and what were their rationales and strategies?

Lastly, we explored the changing nature of these 4 factors over time, with particular attention to the aspect of resistance.

## FINDINGS

### Mexico in Context

Mexico is a large middle-income country with an adolescent population of 23.7 million people in 2015, composing 19% of the country's total population.^32^ In 2009, approximately one-quarter and one-fifth of adolescent boys and girls, respectively, were sexually active, and almost 30% of married and/or sexually active girls aged 15 to 24 years had an unmet need for contraception.^33^^,^^34^ In most states of the country, access to safe abortion services remains restricted.^35^ Because of limited access to sexual and reproductive health services and conservative social factors, national adolescent pregnancy and unsafe abortion rates have been high, albeit with regional variation.^33^^,^^34^^,^^36^ Meanwhile, national attendance rates in Mexico's primary and secondary schools have reached 98% and 80%, respectively, giving school-based sexuality education programs the potential to reach many young people and reduce these risks if implemented and delivered effectively.^37^

National attendance rates in Mexico's primary and secondary schools are very high, giving school-based sexuality education programs the potential to reach many young people.

Mexico is regionally and ethnically diverse and is defined by its constitution as a pluricultural state. Mexican society also has a strong Catholic foundation—with more than 80% of the population nominally affiliated with the church—and strong conservative values on premarital sexual activity, traditional family structures, and inequitable gender norms.^38^ Despite this, the country has a largely secular government, which has promoted progressive policies, and a decentralized system of political authority comprised of 31 independent and autonomous states and the Federal District of Mexico City. These factors contribute to substantial diversity in the nation's state-level policies. For example, while the federal government decides on the content included in curriculum and textbooks, which are delivered to all children and adolescents free of charge, state governments control the content of an extra module in secondary schools. Together, these factors have created challenges to implementing a cohesive national framework for adolescent sexual and reproductive health education, despite demonstrated need.^39^

### How Sexuality Education in Mexico Evolved, 1930s–2010s

**FIGURE 1.**
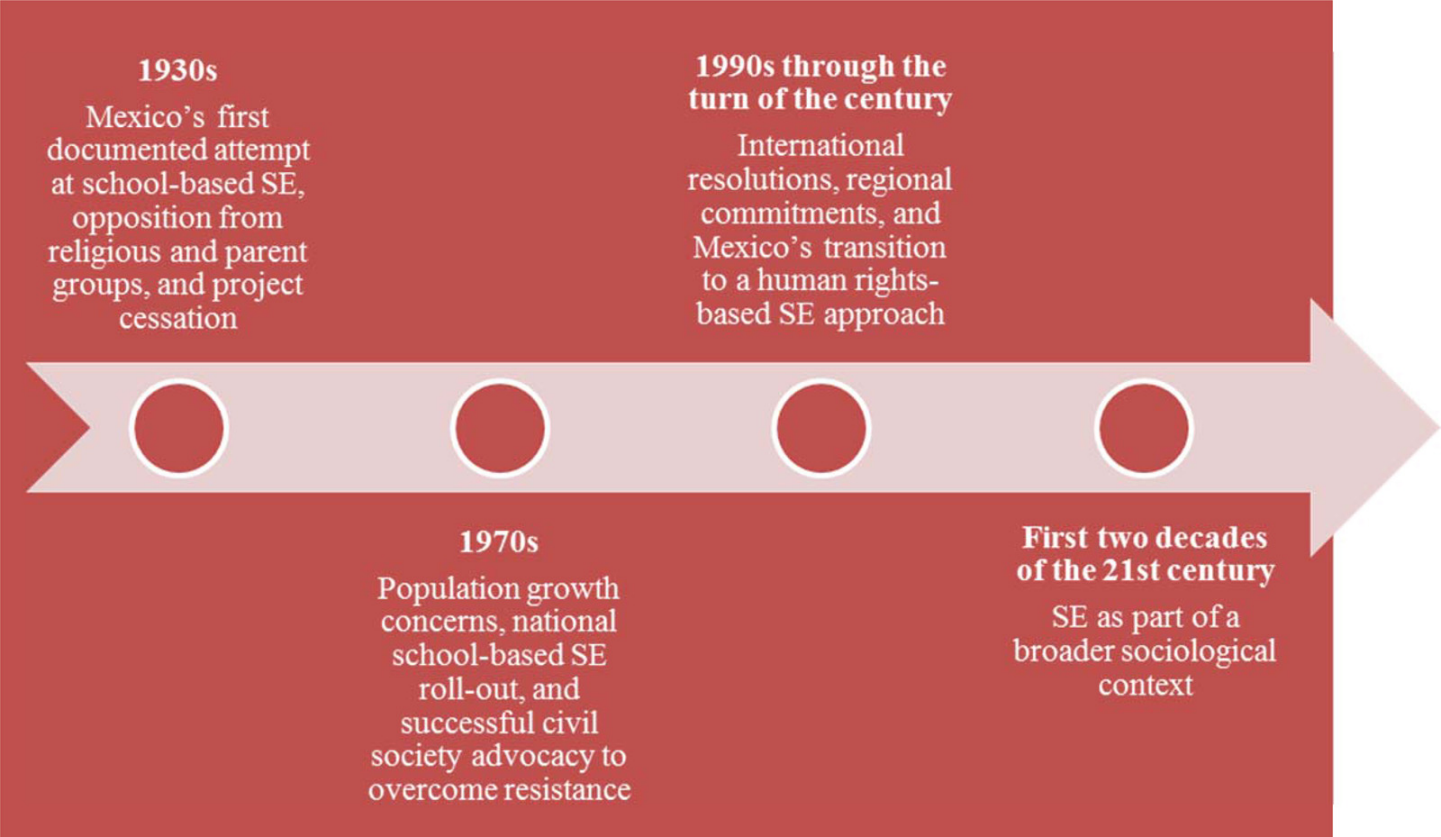
Historical Time Periods of the Evolution of Sexuality Education in Mexico Abbreviation: SE, sexuality education.

Our findings are first organized chronologically by the following 4 historical time periods (Figure 1).

#### 1930s: Mexico's First Documented Attempt at School-Based Sexuality Education

The first documented attempt to introduce sexuality education in primary schools in Mexico City was led by Public Education Secretary Narciso Bassols in 1932.^16^^,^^26^ This initiative, part of his larger vision of a socialist education policy, was driven by sexuality education recommendations from the 1930 Pan-American Congress of the Child, held in Lima, Peru, and aligned with objectives of the Mexican Eugenic Society. The Mexican eugenics movement, prompted by concerns over poverty, internal displacement, and high rates of mortality and illness after the Mexican Revolution (1910–1917), greatly influenced national policies on public health, education, and welfare in the 1930s, especially those related to maternal and reproductive health. The rationale of Bassols's initiative was that children are part of a secular world where schools play a significant role, and, thus, schools are obliged to respond to the rights of children to obtain information on conception, birth, and reproduction.^20^

The first documented attempt to introduce sexuality education in primary schools in Mexico City was led by Public Education Secretary Narciso Bassols in 1932.

Many government officials, academics, and health and education professionals supported the underlying rationale for Bassols's initiative, agreeing that sexuality education is necessary for children to reach their full potential and achieve healthy development.^20^ However, the initiative became conflated with an ongoing conflict between the Catholic Church's assertion of religious authority and the Mexican State's assertion of secular authority.^25^ Supporters and critics of sexuality education both used the media to influence public opinion and lobby stakeholders to support or oppose sexuality education. Strong resistance arose from conservative parent associations of private schools and religious institutions.^31^ Arguments against school-based sexuality education asserted that parents and the family should provide sexuality education, as they were best able to assess the age-appropriate information needs of their children and that only families had the right to provide their children with values regarding sexuality. Many parents also agreed with religious leaders who worried that sexuality education would encourage adolescents to engage in sexual activity.^40^ Concerned mothers gathered for demonstrations and alleged that sexuality education was a communist plot. Male secondary school students protested sexuality education in a movie theatre, arguing that it would corrupt their female classmates, and their arrest prompted a general school strike and street protests.^23^

Arguments against school-based sexuality education asserted that parents should provide such education, as they are best able to assess the age-appropriate information needs of their children.

Soon after the initiative's inception, Bassols was asked to resign—a decision attributed to both his sexuality education initiative and his support for socialist education.^7^ The first official effort for school-based sexuality education in Mexico failed to sustain itself amidst substantial resistance, and the initiative was halted.^23^

#### 1970s: National School-Based Sexuality Education Roll-Out

During the 1970s, policy makers in Mexico became increasingly concerned with demographic trends, including rapid population growth, driving a renewed prioritization of sexuality education. Government officials introduced numerous policies, including a modification of the Mexican Constitution to protect the individual's right to decide the number and spacing of their children, the creation of the National Population Council, and the introduction of a family planning policy in the Secretariat of Health. These changes enabled the government to declare school-based sexuality education the educational foundation for population policies. In response, sexuality education was introduced into national primary and secondary education curricula in the early 1970s and integrated into several mandatory courses, including biology and civic education.^22^ The new sexuality education content was rooted in human biology, and reinforced conservative values that recognized only heterosexual unions and marriage as acceptable spaces for sexual practices.^19^^,^^21^^,^^23^ Chapters on puberty, menstrual cycles, pregnancy, and childbirth were added to primary school textbooks, and information on prevention of unintended pregnancy and sexually transmitted infections was added to secondary school textbooks. These textbooks were distributed free of charge to all children in public schools. Primary and secondary school teachers received preservice training at teacher training colleges, such as the National Pedagogical University (Universidad Pedagógica Nacional), to incorporate the textbooks' content into the curriculum.

During the 1970s, policy makers in Mexico became increasingly concerned with demographic trends, including rapid population growth, driving a renewed prioritization of sexuality education.

Despite the relatively conventional nature of the sexuality education content, conservative sectors, arguing for adherence to traditional and cultural norms, fervently resisted the school-based sexuality education program.^22^ They asserted again that it was the parent's right to teach values about sexuality to their children, and that sexuality education was not an appropriate topic to be taught in schools. For example, parents and teachers were opposed to discussion of masturbation and accused sexuality education of promoting socialist ideas.^23^ At times, the backlash reached violent expression: book burnings and calls to tear the offending pages from textbooks occurred in Aguascalientes and San Luis Potosi, 2 of the most conservative and religious states.^19^

In contrast to the opposition from conservative political parties, parent associations, and Catholic sectors, Mexico's secular government supported the integration of sexuality education content into the education curriculum.^23^ The curricula development team, with the approval and support of then-Secretary of Education Victor Bravo Ahuja, organized a tour of numerous cities to inform communities about the curricular changes and met with opposition groups, particularly in areas where major protests had occurred. Additional support for school-based sexuality education was led by organized civil society and NGOs, such as the Mexican Association for Sex Education, founded in 1972. Through the years, the number of NGOs directly or indirectly involved with the promotion and delivery of sexuality education grew substantially, largely influenced by the emergence of the HIV/AIDS pandemic in the 1980s. By advocating with uncertain or critical government officials and nongovernmental stakeholders about the rationale and characteristics of sexuality education, these groups reinforced the government's sustained support for sexuality education. As a result, sexuality education content was retained in the national curriculum and textbooks with only minor changes until the mid-1990s.

#### 1990s: Mexico's Transition to a HumanRights-Based Sexuality Education Approach

Building on progress made in the 1970s and 1980s, Mexico affirmed its commitment to sexuality education in the 1990s alongside resolutions of the International Conference on Population and Development in 1994 and the Fourth World Conference on Women in 1995. Driven by ongoing organized civil society advocacy efforts and growing evidence of sexuality education's benefits for young people, Mexico issued a reform on the General Law of Education in 1993 to include clauses in support of the goals of sexuality education ^22^^,^^40^:

***Article 7.-*** *The education imparted by the State … will … create conscience of the preservation of health, family planning and responsible paternity, without impairment of freedom and absolute respect of human dignity. …*

***Article 8.-*** *The rationale guiding education that the State and its decentralized organisms impart … will be based on results of scientific progress, fighting ignorance and its effects, servitude, fanatics and prejudice.*

As a result of this reform, primary school textbooks on natural science, civics, and ethics were expanded to include social, emotional, and ethical aspects of sexuality, including information on gender, sexual rights, and pleasure. While states controlled the content included in the extra module in secondary schools, inclusion of sexuality education content in national textbooks and curricula resulted in the most progressive education curricula in Mexico's history to date.

Driven by ongoing organized civil society advocacy efforts and growing evidence of sexuality education's benefits for young people, Mexico issued a reform on the General Law of Education in 1993 to include clauses in support of the goals of sexuality education.

The policy reform met fierce resistance from Catholic Church authorities and organizations, such as the National Union of Parents of Families (Unión Nacional de Padres de Familia [UNPF]) and the National Pro-Life Committee (Comité Nacional Pro-Vida) (Box).^29^^,^^31^ Groups of parents deemed the new textbooks “pornographic” and “perverse.”^25^ While most of the resistance manifested in local protests, it was also communicated through the media, with debates about sexuality education featured on television and radio.^23^

BOX **Important Players in the Opposition to Sexuality Education in Mexico**

- The **National Union of Parents and Families** was created in 1917 as a conservative group opposed to the Constitution's liberal and secular articles. In particular, it opposed the third article that guarantees secular education and, later, the provision of free textbooks and sexuality education to school children. The objective of the union was to allow parents to demand respect for their rights, including the right to educate their children according to their principles and values.^17^ Demonstrations by the union against sexuality education date back to 1934.^19^
- The **National Pro-Life Committee** was founded in 1978 as an anti-abortion NGO with links to the Catholic Church. This organization played a major role in resisting legislative attempts to decriminalize or liberalize abortion in Mexico, as well as opposing same-sex marriage, LGBTI rights, and sexuality education.^20^
- The **National Front for the Family** is an alliance of more than 1,000 civil society institutions nationwide, which uses Catholic principles to defend the sacred institution of matrimony between man and woman and the natural family as the basis of society. It was created in 2016 to oppose the president's initiative to recognize and legalize same-sex unions. The alliance is gathering support for a petition for constitutional reform introduced to the Senate on February 2016 by ConFamilia (Consejo Mexicano de la Familia). The initiative, which has gathered more than 200,000 signatures, calls for the recognition and protection of the family entity and unions of men and women and the guarantee of parents' right to choose the kind of education their children receive. Lastly, it demands that the impact on the family entity be evaluated for all laws and policies.^18^

Civil society's support for sexuality education from the 1970s was strengthened by the numerous organizations formed in the 1980s in the wake of the HIV/AIDS pandemic.^23^ In the 1990s, these NGOs formed 2 large networks—the Mexican Federation of Sexology and Sex Education (FEMESS) and Democracia y Sexualidad (DEMYSEX)—that linked more than 300 organizations, including MexFam, an International Planned Parenthood Federation affiliate. They collaborated to use print media, such as newspapers and magazines, and audiovisual media, such as television and radio, to build favorable public opinion and community support. Through activities including press releases, conferences, and academic events, the networks were able to build community support and successfully expand and sustain the new human rights-based sexuality education content.^41^^,^^42^

Civil society's support for sexuality education from the 1970s was strengthened by the numerous organizations formed in the 1980s in the wake of the HIV/AIDS pandemic.

#### First 2 Decades of the 21st Century: Sexuality Education as Part of a Broader Sociological Context

In the first 2 decades of the 21st century, responses to sexuality education became increasingly driven by linkages to related movements, including HIV prevention, adolescent pregnancy prevention, and lesbian, gay, bisexual, transsexual, and intersex (LGBTI) rights promotion. In 2008, Mexico City was home to the 17th International AIDS Conference and the first Meeting of Ministers of Health and Education of Latin America and the Caribbean (LAC). The latter meeting brought together 30 Ministers of Health and 26 Ministers of Education, or their personal representatives, from the LAC region, who collectively committed to implement sexuality education and sexual health promotion programs in their countries in order to promote concrete action for HIV prevention among young people. The main outcome, the Ministerial Declaration “Preventing through Education,” included goals to reduce by 50% the number of adolescents and young people who lack access to sexual and reproductive health services and to reduce by 75% the number of schools that failed to institutionalize sexuality education. This declaration, along with a progressive series of implementation evaluations, was an important tool to help strengthen and revitalize government commitment to sexuality education, not only in Mexico, but throughout Latin America and the Spanish-speaking Caribbean.^43^

While the Ministerial Declaration and other advocacy and programmatic efforts improved the Mexican government's official commitment to sexuality education, they had a limited impact on preventing adolescent pregnancy. Despite major declines in total fertility rates, adolescent birth rates have not decreased to the same extent and the percentage of registered births to adolescent mothers under 20 years old has continued to increase—from 16.9% in 1994 to 18.1% in 2015.^44^ President Enrique Peña Nieto prioritized addressing this concerning trend by developing an intersectoral National Strategy for Adolescent Pregnancy Prevention (ENAPEA) in 2015.^45^ Within this strategy, the Ministry of Education developed a sectoral agenda for comprehensive sexuality education to ensure such education was rooted in evidence-based curricula that considered the biological, psychological, social, cultural, economic, and political aspects of sexuality with respect to human rights and dignity. This measure aimed to improve the quality of education and to ensure access to, continued enrollment in, and completion of schooling as a means of preventing unwanted/unplanned adolescent pregnancies.^46^

Despite major declines in total fertility rates, adolescent birth rates have not decreased to the same extent and the percentage of births to adolescent mothers under 20 years old has continued to increase.

In 2015, resistance to the adolescent pregnancy prevention initiatives took the form of a digital petition platform to revise the ENAPEA. In this platform, citizens expressed concerns about the strategy, arguing that it was based on distribution of contraceptive methods and condoms to adolescents in schools and public places without parental approval, encouraged early adolescent sexual activity by incorporating discussions on pleasure, and promoted abortion as a family planning method. Furthermore, the petitioners argued that the strategy failed to consider values of love, compromise, and responsibility in expressing sexuality and did not adequately promote abstinence until marriage. This particular petition was promoted by 7 organizations, including the UNPF, gathering over 14,000 signatures by 2016.^47^

Additionally, sexuality education experienced a new wave of opposition driven by the current administration's promotion of LGBTI rights. On the 2016 National Day against Homophobia, President Peña Nieto convened LGBTI groups and other NGOs, including FEMESS, to launch an initiative calling on members of Congress to modify the civil code and other laws to guarantee the right to same-sex marriage and access to changes in gender identity, and establish equality for adoption, among other initiatives.^48^ As a result, Secretary of Public Education Aurelio Nuño promised to revise the current sexuality education curriculum to include sexual diversity by 2017.^49^ Protests from conservative voices, such as the National Catholic Bishops' Conference and the UNPF, arose quickly and a new alliance of resistance formed in May 2016: the National Front for the Family (Box).^30^^,^^50^ This opposition group used social media networks to organize several nationwide protests to influence public opinion against the presidential initiative and the revision of the national textbooks. They convened massive demonstrations and attracted substantial external sources of technical and financial support for opposition to sexuality education to a degree that previous waves of resistance had not experienced.^51^^–^^53^ Many believe that the opposition's critical reaction to the President's decision in favor of inclusion of sexual diversity largely influenced the governor elections in 2016, which resulted in great losses for the ruling party. In November 2016, the initiative for constitutional reform to guarantee rights for the LGBTI community was rejected by Congress.^54^

During this recent period of opposition, NGOs supporting sexuality education in Mexico persisted in leading research, technical assistance for teacher trainings and production of educational materials, and, especially, advocacy for sexuality education's inclusion in formal and non-formal curricula. The Mexican Association for Sexual Health (AMSSAC) continued to conduct nationwide training programs for teachers, which they had initiated as early as 2012, to complement broader violence prevention efforts.^51^ The National Population Council and the Mexican Institute for Radio ran a series of media campaigns on adolescent sexual and reproductive health, which included phrases such as “The responsibility is yours. Informing yourself is your right. Protect yourself.” and “Informed, free, and safe sexuality avoids surprises which can alter your future.”^55^^,^^56^ The DEMYSEX alliance led advocacy efforts at the state level. In 2016, supporters of sexuality education achieved a major victory in a Supreme Court of Justice ruling that established children and young people's right to comprehensive sexuality education and contraception as a component of their basic human right to the highest possible level of physical and mental health. According to government officials, including the Secretary of Health, and in the view of supportive NGOs, this ruling offers many opportunities for the defense and continuation of comprehensive sexuality education programs and actions.^52^^,^^53^ Accordingly, on International Population Day in 2017, the National Population Council explicitly called for strengthening of adolescent sexuality education^.57^

NGOs supporting sexuality education in Mexico persisted in leading research, technical assistance for teacher trainings and production of educational materials, and advocacy for sexuality education's inclusion in formal and non-formal curricula.

### How Drivers, Responses, Support, and Resistance Impacted Mexico's Delivery of School-Based Sexuality Education

The following sections analyze the evolution of sexuality education in Mexico by extracting crosscutting themes within the 4 thematic aspects: drivers, responses, support, and resistance.

#### Drivers

The drivers in the evolution of efforts toward national school-based sexuality education in Mexico have changed over time. Starting from a socialist education project in the 1930s, based on recommendations from the Mexican Eugenics Society and the Pan-American Congress for the Child, the drivers shifted to concerns about rapid population growth in the 1970s, to commitments toward international treaties and regional agreements in the 1990s, and, finally, to adolescent pregnancy prevention targets, protection of LGBTI rights, and efforts to address discrimination in the 21st century. In 2016, human rights movements and political momentum made a substantial impact, culminating in the acknowledgment and inclusion of adolescents' right to sexuality education and contraception as a basic element of a child's human rights, as recognized by the 2016 Supreme Court ruling.

#### Responses

The responses prompted by these drivers varied, greatly influenced by the social, economic, and political contexts of each time period. Mexico's first major response in this analysis was the initiation of a national school-based sexuality education initiative in the 1930s, followed later by inclusion of sexuality education content in textbooks and curricula for all public primary and secondary schools in the 1970s. The government has responded to the evolving drivers by updating and expanding the curricula and programs toward a comprehensive approach to sexuality, albeit not in a continuous or consistent manner. The government also recognized the potential benefits of delivering sexuality education to children and adolescents as a strategy to prevent adolescent pregnancy and to eliminate different forms of discrimination. Finally, the recent Supreme Court ruling offers promising opportunities for the defense and continuation of sexuality education in Mexico. Mexico's decentralized power structure, meanwhile, has influenced the degree to which sexuality education has been introduced and supported in each state. While federal authority defines the curricula of national textbooks and compulsory courses, which include sexuality education content as described previously, in order to ensure standards of quality in the country, state-level authorities control the content selected for the extra module in secondary school curriculum. To that end, state authorities have a degree of autonomy in meeting supplementary educational needs and administering educational services tailored to the specific demands of each state. It is important to note, also, that the varied degree of progress in implementing sexuality education at the state level is also related to the heterogeneous characteristics, political interests, and levels of influence of supporters and opposition in each state.

The government recognized the potential benefits of delivering sexuality education to children and adolescents as a strategy to prevent adolescent pregnancy and to eliminate different forms of discrimination.

#### Support

Support for delivery of school-based sexuality education has existed in Mexico since the 1930s. Government officials have championed many of the sexuality education initiatives, resulting in legal and policy changes and educational reforms. Additionally, growing evidence on the benefits of comprehensive sexuality education for positive sexual and reproductive health outcomes of young people has proven useful for pushing forward the agenda for national school-based sexuality education. Several organized civil society organizations and NGOs have been instrumental in advocating, promoting, and delivering sexuality education programs in Mexico. Since the 1990s, individual organizations have joined forces to support sexuality education as coalitions of organizations engaged in collaborative work.

#### Resistance

**FIGURE 2.**
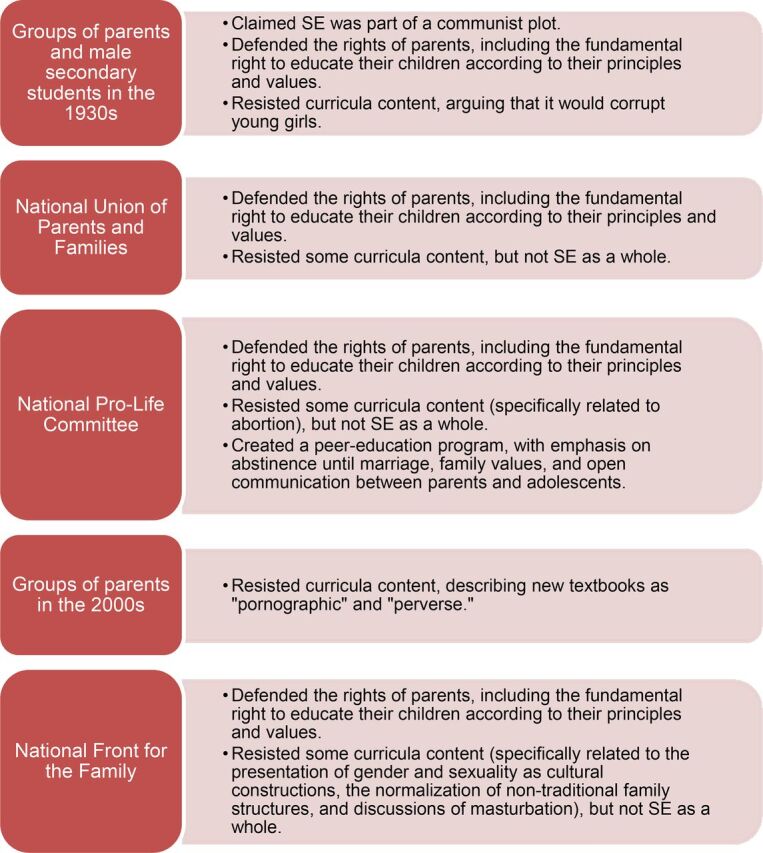
Opposition Groups and Their Rationale for Resistance to Sexuality Education

Resistance to delivery of school-based sexuality education was encountered during each historical time period from conservative parents' unions, the Catholic Church, and other faith-based organizations. From the review of 3 organizational websites for opposition groups (Figure 2), it is evident that organizations oppose sexuality education for distinct reasons, albeit with common threads. For the most part, they have not completely rejected the concept of sexuality education; instead they object to its place in schools and fight to uphold familial and parental authority in their children's education. These groups believe that school-based sexuality education could have harmful consequences for the lives of children and adolescents, such as the promotion of early and risky sexual activity, masturbation, or homosexuality. Most of the identified groups had ties to the Catholic Church and, thus, defended religious values.^29^^–^^31^ In recent years, resistance to sexuality education has been linked with movements against abortion and same-sex marriage. The organizations that resist these initiatives formed united coalitions, rallied thousands of people into massive demonstrations, led intensive lobbying against related government initiatives, and used mass media to reach the population at large.^30^

For the most part, organizations opposing sexuality education have not completely rejected the concept but instead object to its place in schools.

## DISCUSSION

The chronologic and thematic analysis of the evolution of sexuality education in Mexico reveals determined support for school-based sexuality education from a range of players in the 4 historical time periods described in this article. Specifically, the commitment of Mexico's secular government to include sexuality education in public school curricula and textbooks and to continuously review and expand the curricula has been instrumental in its longevity. The findings also reveal that opposition to sexuality education has existed since its first introduction in schools and persists today, and that rationales for opposition have remained largely consistent. Some of the opposition's concerns are well-intended; parents, for example, are worried for their children and are concerned that sexuality education programs are sharing messages that conflict with their values. However, other opposition groups have included false accusations to discredit and slander efforts toward national implementation of quality school-based sexuality education. Opposition to sexuality education, but also to many human rights issues including abortion and LGBTI rights, rose steadily during the time period analyzed, with a growing range of more organized and well-financed actors. As sexuality education has become linked with these issues over time, it has attracted additional backlash and resistance.

Since the first implementation of sexuality education in Mexico, the government's response has been inconsistent and has not employed systematic strategies to build community support for sexuality education. Additionally, little dialogue has occurred between supporters of sexuality education and its opposition; what communication has occurred has been facilitated through the media. In particular, most of the organizations belonging to the FEMESS and DEMYSEX networks, which represent more than 100 organizations, prefer not to work directly with opposition groups, arguing that these groups are usually not willing to discuss or move from their extreme positions. Instead, supporters have focused on advocating with political stakeholders in order to attain further support for sexuality education within the government.

We found that the rationales used by organizations opposed to school-based sexuality education in Mexico are similar to those in other countries in the region. In Panama, a draft law for establishing a normative baseline for safeguarding and promoting sexual and reproductive health was introduced in 2014 and backlash included protests by Catholic and Evangelical churches and other groups.^58^ Similarly, in Colombia, conservative actors, including Red Familias and members of the Catholic Church, demonstrated against initiatives from the Ministry of Education, United Nations agencies, and NGOs to rid schools of discrimination. These initial protests ultimately resulted in massive protests in Bogota and other major cities. The emergence of coalitions of resistance to sexuality education in Mexico and other Latin American countries is a pattern that has been seen elsewhere, including in protests against numerous other movements such as LGBTI rights, same-sex marriage and adoption, and abortion—that often include false accusations about the use of sexually explicit materials in school-based sexuality education curriculum. A common feature in these protests is the condemnation of the so-called “gender ideology,” a term derived by conservative groups that is a misconstruction of gender theory. According to those who use the term, gender ideology proposes to eliminate the differences between men and women, including biological differences. These groups adhere to a rigid binary view of sex that male and female nature is set and that there is a “natural family,” based in heterosexual marriage and the procreation of children. The influence of religious conservatism in the region is also illustrated in the case of Brazil, where conservatism has gained influence in government, endangering progress made by the country toward the protection and promotion of human rights in general, and women's sexual and reproductive health and rights in particular.^59^ The similarities of rationales and strategies from a review of the coalitions' websites suggests their efforts are a supranational movement financed by substantial international funds.^60^

The commitment of Mexico's secular government to include sexuality education in public school curricula and textbooks and to continuously review and expand the curricula has been instrumental in its longevity.

While this research did not assess coverage and quality of sexuality education in Mexico, a survey of a nationally representative sample of almost 4,000 students ages 15 to 18 years in urban and rural areas identified that the proportion of adolescents receiving sexuality education varied greatly depending on grade level.^59^ Furthermore, due to Mexico's decentralized government, the extent to which sexuality education has been included in extra modules, and the quality and fidelity with which it has been implemented, was found to be highly variable by state.^59^ Students reported that the curricula were often incomplete and taught unevenly throughout the school year, and that the methodologies teachers used to facilitate better uptake of knowledge and skills should be improved.^61^ These findings indicate that alongside efforts to create an enabling environment for sexuality education, attention needs to also be given to ensuring quality and fidelity of sexuality education.

### Limitations

While this article is largely based on the experience of an expert with more than 50 years in the field of sexuality education in Mexico, her testimonies were complemented with evidence from peer-reviewed literature and, given the limited number of peer-reviewed publications on this subject, numerous other data sources, including gray literature, periodic publications, and website content. Additionally, as described earlier, this analysis does not restate the evidence base for sexuality education, nor does it assess the program's coverage, quality, or fidelity, or the impact of the program on knowledge, behavioral, or health outcomes. While the article does not present recommendations on best programmatic practices, it does describe one country's experiences in implementing and sustaining sexuality education over time.

## CONCLUSION

Sexuality education continues to be supported and resisted by different groups within Mexico's government, NGOs, and organized civil society. In the last 2 decades, opposition to school-based sexuality education has become more organized and has gathered greater numbers of constituents and resources to target not only sexuality education but also sexual and reproductive health and rights in general. In response, a number of recommendations can be made to ensure the future delivery of school-based sexuality education in Mexico. Firstly, the review revealed that alliances for sexuality education can and should be built between academic, government, organized civil society, and NGO champions to organize efforts and work strategically and cohesively to respond to opposition. Advocates for school-based sexuality education must capitalize on the momentum of the recent Supreme Court victory that established children and young people's right to comprehensive sexuality education and contraception, while also building strategies to engage diverse communities of Mexico and confront resistance from well-organized opposition alliances and networks. Secondly, supporters must learn about the opposition and its networks, perspectives, and methods, and develop strategies tailored to specific groups and contexts. Thirdly, the defense of the secular state must be sustained, as it has proven to be one of the best safeguards to efforts to sustain sexuality education in a complex and diverse country such as Mexico. Lastly, greater attention could be given to employing systematic strategies to build community support for sexuality education.

Alliances for sexuality education can and should be built between academic, government, civil society, and NGO champions.

This analysis of the evolution of sexuality education in Mexico shares one example of responses to resistance in a changing social and political context and can inform other countries' efforts to consider the drivers, response, support, and resistance to sexuality education that may be present in their own contexts. In particular, the movement for sexuality education in the entire Latin American region can learn from Mexico's experience due to the similarities in rationales and strategies of resistance, such as religious conservatism.

## Notes

**Peer Reviewed**

**Funding:** This work was funded by the UNDP-UNFPA-UNICEF-WHO-World Bank Special Programme of Research, Development and Research Training in Human Reproduction (HRP), a cosponsored program executed by the World Health Organization (WHO). ECV was engaged by WHO to produce a working draft of this paper.
